# A Rare Medical Dilemma: Presentation and Management of Placental Polyp

**DOI:** 10.7759/cureus.12259

**Published:** 2020-12-24

**Authors:** Raghad Alhussami, Fatma Noorwali, Ghadah Ibrahim

**Affiliations:** 1 Obstetrics and Gynecology, King Abdulaziz Medical City - Jeddah, King Saud bin Abdulaziz University for Health Sciences, Jeddah, SAU; 2 Obstetrics and Gynaecology, King Abdulaziz Medical City - Jeddah, Jeddah, SAU

**Keywords:** placental polyps, retained fragment of placental tissue

## Abstract

Placental polyp is one of the rare diseases that affect women after parturition or abortion. In this series of case reports, we aim to demonstrate the presentation, imaging findings, and management of these cases of placental polyps, which were presented to our institution. These cases vary in age and clinical findings. Our objective is to shed light on this disease, which could be initially disregarded. Also, we analyze the screening modalities that may aid in reaching the appropriate diagnosis, and possible treatment options.

## Introduction

A placental polyp is defined as a retained fragment of the placental tissue within the endometrial cavity after parturition or abortion, resulting in the formation of polypoidal mass in the uterus. Organized villi and decidua, in addition to degenerated clots and regenerated endometrium, are seen histologically [1].

Although many conditions cause postpartum hemorrhage, a placental polyp is considered rare [1]. Epidemiologically, placenta polypoid masses are found in less than 0.25 % of all pregnancies. Interestingly, hypervascular placental polyps that are associated with severe hemorrhage are found in 6% of those cases. Women who have placental polyps may present within a few days to weeks, either after a therapeutic abortion or after spontaneous delivery, and even more rarely seen after a spontaneous abortion [2]. 

This condition is usually diagnosed by using color Doppler ultrasound, computed tomography (CT), or magnetic resonance imaging (MRI); however, the best diagnostic method for placental polyps is to do diagnostic hysteroscopy and polyp resection with the histopathological examination, which is also considered therapeutic [3].

## Case presentation

Case 1

A 31-year-old woman G4P3+1 presented with a history of continuous vaginal bleeding for six months duration post complete abortion. The patient was diagnosed as a case of incomplete abortion in another hospital, where she was given one dose of misoprostol and passed the tissue (the histopathology was not documented). The patient had a follow-up due to her persistent vaginal bleeding and was found to have a low hemoglobin level, for which she was given a blood transfusion. Ultrasound was done twice before presentation to our hospital. The first ultrasound showed a bulky uterus with an endometrial thickness of 18 mm, no intrauterine tissue, and normal adnexa. The second ultrasound showed a bulky uterus with an endometrial thickness of 15 mm, well defined cystic lesion measuring 5 mm inside the uterine cavity, which appears as a gestational sac of the retained product of conception.

The patient presented to our clinic six months later with continuous heavy vaginal bleeding with passage of clot. She denies any passage of tissue or abdominal pain. She had symptoms of anemia but no symptoms of infection. Her examination was unremarkable, apart from mild vaginal bleeding. Her pregnancy human chorionic gonadotropin (hCG) was negative. Endometrial biopsy was done to prove the presence of trophoblastic tissue. The endometrial biopsy showed benign weakly proliferative endometrium with focally embedded necrotic chorionic villi with no hyperplasia or dysplasia identified.

Ultrasound in our hospital showed an endometrial thickness of 0.4 cm. There was an endometrial polyp 1.5x2.0 cm with a large single feeding artery. Minimal free fluids in the pouch of Douglas were noted. Ultrasound images for this case were not available as they were not uploaded to our hospital system.

The patient underwent hysteroscopy resulted in a normal endometrial cavity, and in the right corner, there was irregular fungating firm necrotic chorionic villi, typical old white trophoblastic projection, which was resected, and the patient started to bleed. The bleeding was difficult to control by cauterization with a resectoscope**. **So, Foley’s catheter size 12F was inserted in the cavity, and 15mls of saline was used to inflate the balloon. Six hours later, the balloon was deflated and removed after confirming that there was no active bleeding. The patient was discharged on the next day in stable condition with no bleeding.

Two weeks later, the patient was seen in the outpatient clinic - there was no bleeding, and the histopathology finding was prominent necrotic retained products of conception (chorionic villi), background benign proliferative endometrium, no hyperplasia, and no dysplasia.

Case 2

A 40-year-old woman G2P2+0 with a previous history of two Cesarean sections presented with a complaint of continuous vaginal bleeding for three months after elective Cesarean section with the passage of small tissue. The bleeding was not associated with abdominal pain, fever, or foul-smelling vaginal discharge. The examination was unremarkable, with a normal size of the uterus.

**Figure 1:**
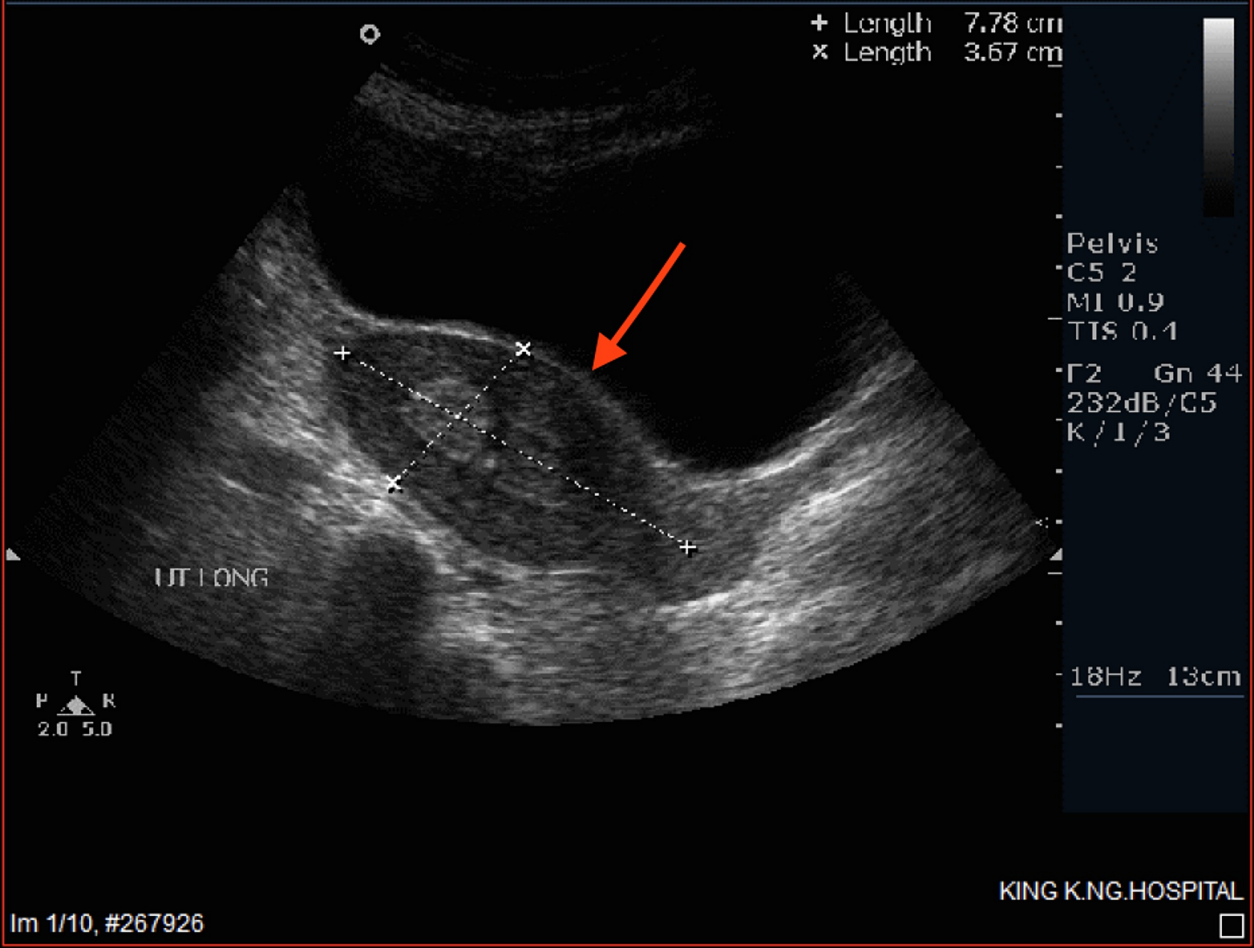
Ultrasound showing endometrial polyp measuring 1.5x2 cm

Her pregnancy test human chorionic gonadotropin (hCG) was negative. Hemoglobin was 11.5 gm/dL. Ultrasound showed uterus 7.5x4.3 cm with thickened endometrium. Echogenic heterogeneous structure mass was seen that measured about 3.6x1.7 cm with no vascularity orcystic changes with minimal free fluids most likely product of conception​​​​​​​ (Figure 1).

The patient was counseled regarding the condition. Hysteroscopy and resection were done, and tissue was sent for histopathology that revealed benign secretory endometrium with a necrotic retained product of conception associated with acute and chronic inflammation, underlining endometrium showed no atypia and no hyperplasia.

A few weeks later, there was no bleeding, and contraception started.

Case 3

A 21-year-old woman G1P0+1, gestational age (GA) 10 weeks with a history of hypothyroidism presented during coronavirus disease (COVID-19) closure with a history of missed incomplete abortion. She was managed with misoprostol, and she developed severe bleeding, due to which she was taken for emergency evacuation. She had curettage, not suction, and the bleeding persisted for two months. The bleeding was minimal without clots, fever, abdominal pain, or foul-smelling vaginal discharge. Upon examination, the patient was conscious and oriented. Her vital signs were within normal. Abdominal and per vaginal exams were unremarkable with a normal size of the uterus.

**Figure 2:**
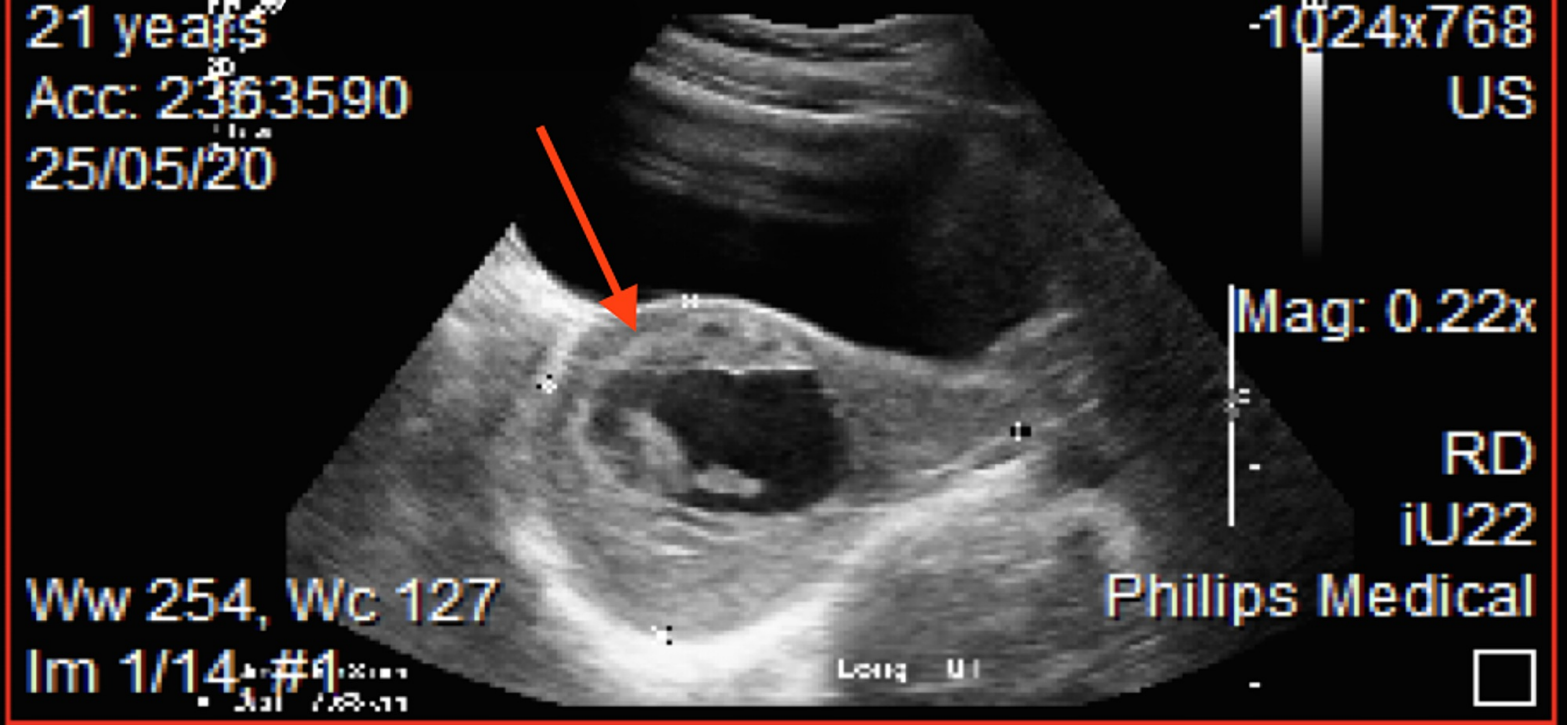
Retained tissue with positive Doppler

Her pregnancy test human chorionic gonadotropin (hCG) was negative. Ultrasound showed heterogeneous thickened endometrium with nidus measuring about 2.0x1.2 cm. There is evidence of bridging vessels at this site. The endometrial wall measured 1.4 cm (Figure 2).

**Figure 3:**
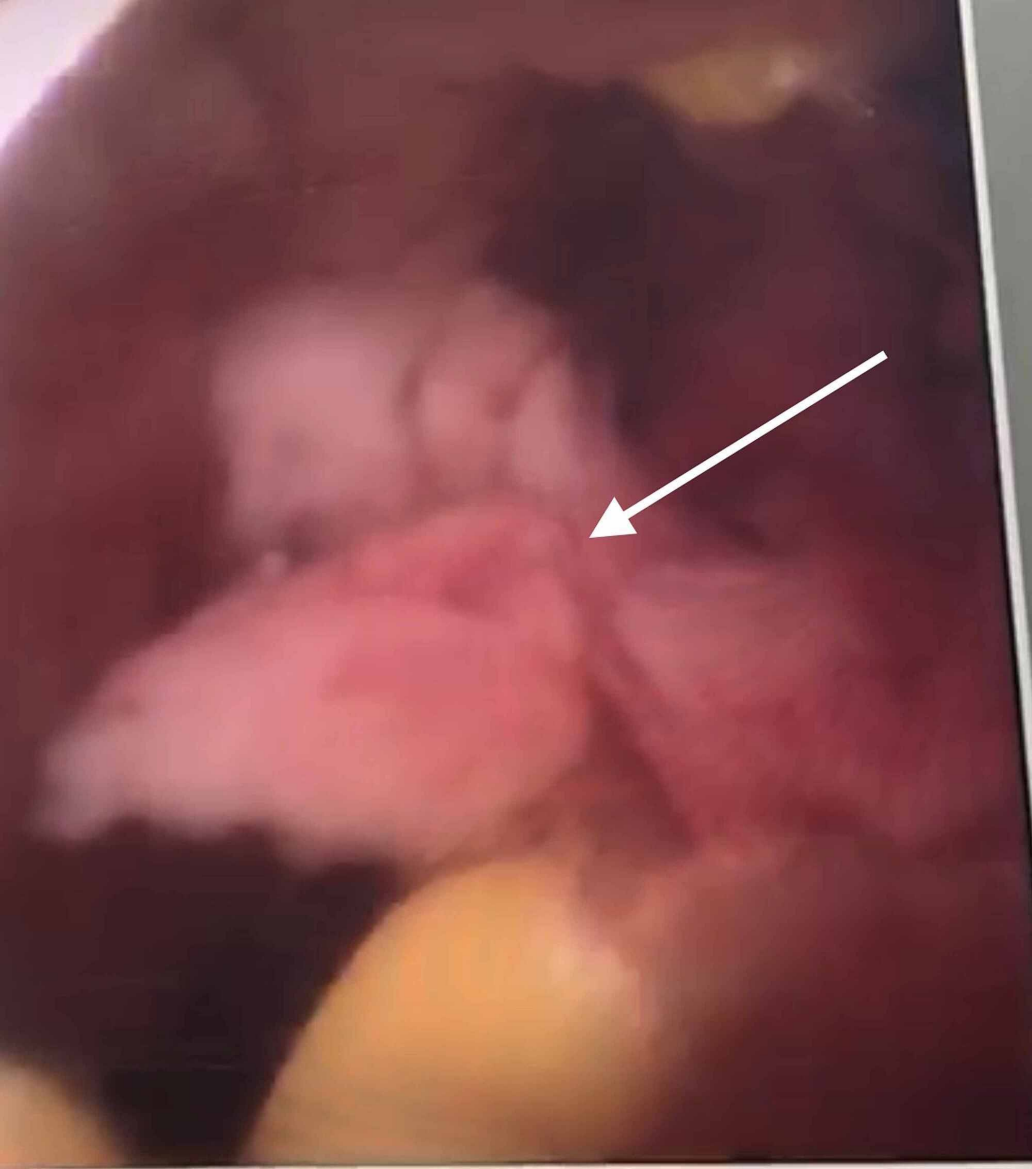
Retained product of conception

**Figure 4:**
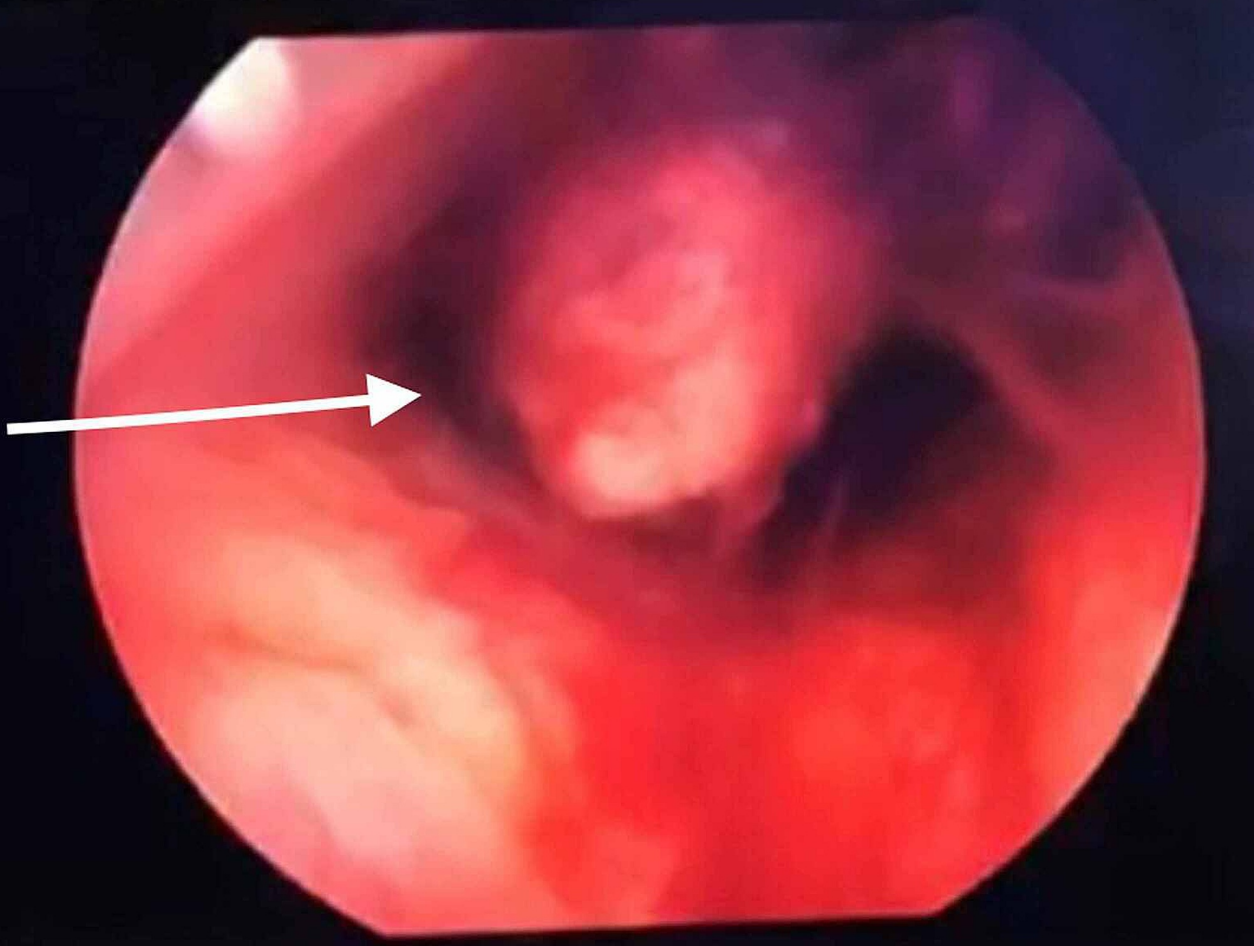
Retained product of conception

The patient was admitted and was planned for diagnostic hysteroscopy and resection of the retained product of conception if needed (Figures 3-4).

The patient was given 400mcg of misoprostol for cervical ripening and then underwent hysteroscopy, where a placental polyp was seen and resected using a 10 mm hysteroscope. Foley’s catheter was left in situ to prevent bleeding for 12 hours. Intraoperative finding during hysteroscopy, we found normal vagina, cervix, normal-looking endometrium, the long endometrial polyp was seen hanging from anterior uterine wall 3x3 cm, and both ostia are normal. The specimen was sent for histopathology, and the results came back as a retained product of conception​​​​​​​ (RPOC). She was kept on oral contraceptives​​​​​​​ (OCP’s) postoperatively.

She was seen a few days later and advised to start combined oral contraceptives and was given an outpatient department ​​​​​​​(OPD) follow-up after two weeks.

## Discussion

A placental polyp is defined as a pedunculated placenta or chorionic fragment retained for an indefinite period of time as the residua of an incomplete abortion or term pregnancy [4]. 

In the past, most cases were treated with hysterectomy, usually performed because of intractable bleeding unresponsive to or exacerbated by dilatation and curettage. The placental polyp is considered to be one of the rare diseases with a low incidence of occurrence; it was estimated to be found around 1 in 40,000-60,000 deliveries [5]. 

The placental polyp is usually categorized into one of two types based on the time of its occurrence: acute and chronic placental polyps. Usually, placental polyps that occur before four weeks postpartum are considered acute, which is the more common type. Any placental polyp that occurs later than that is considered chronic. Even though the acute type is found in the majority of women diagnosed with a placental polyp, some cases reported occurrence as late as 20 years postpartum or even five years postmenopause. Interestingly, such tissue can remain in situ for many years in a relatively well preserved and recognized state [5]. 

There are numerous factors that are thought to cause placental polyp. However, two main theories could explain the pathogenesis. The first is that due to the atonicity and thin wall of the cornual or fundal myometrium, the placenta that is adherent to the wall would be more complicated to extract. The second may be attributed to the fact that placental polyps originate from either partial or focal areas of placenta accrete, where the adherence of the villi over the myometrium occurs due to defective decidua. In the presented cases, the microscopic results showed invasion of trophoblastic villi into the base of the myometrium. Unfortunately, there is still no clear cause for the risk factors why they occur in the first place [5]. 

Clinical features depend on the duration of the polyp, including postpartum bleeding, menometrorrhagia, vaginal spotting, lower abdominal pain, and mass protruding from the external os [4, 5]. Usually, it is difficult to be diagnosed, and with persistent bleeding, they used to end up with hysterectomy and diagnosed in the histological specimen. 

In our cases, pregnancy tests were negative, as documented in previously reported cases [4]. However, reported cases documented that survived villi were able to produce low levels of human chorionic gonadotropin (hCG) [5]. If positive, a more serious disease should be excluded [4]. 

Various imaging techniques, including ultrasonography with color Doppler signal, power Doppler imaging, and magnetic resonance imaging (MRI), have been described, but none has ever made a preoperative diagnosis of the placental polyp. The definite diagnoses in previously reported cases were ascertained by tissue histopathology, obtained inadvertently after curettage or hysterectomy due to other diagnoses of bleeding. By nature, placental polyps contain abundant blood supply, more accurately detected by power Doppler imaging or MRI than by the conventional color Doppler signal [5, 6]. These sophisticated tools may be useful for planning further treatment. 

Later therapeutic reports endeavored to preserve fertility, such as conservative vaginal resection [7], selective transarterial embolization before hysteroscopic removal [8], and even methotrexate administration instead of surgery [9]. 

Given the previously mentioned information, we can see that the management may differ from one case to another. In our first two cases, the patients presented with a chronic placental polyp. However, it was difficult to diagnose in the first case as the patient presented to our hospital after being investigated twice in another hospital. Unfortunately, the case is more than 10 years old, and thus ultrasound imaging was not uploaded to our hospital system. The second case was investigated properly from the first presentation to our hospital. In both cases, ultrasound was key in giving the provisional diagnosis, and hysteroscopy was used to diagnose and manage placental polyp. Histopathology was used for confirmation of the diagnosis. However, the third case had more of an acute presentation. The patient was admitted and was investigated. One of our differential diagnoses that should always be considered is an arteriovenous malformation, as they can present similar to placental polyp [1].

The implementation of Foley’s catheter in controlling bleeding showed great efficacy in our cases. This could aid in both localizing and controlling the site of bleeding. Preservation of the uterus should always be one of the main goals nowadays, especially with the availability of more advanced and less invasive options for management and more expertise in the use of hysteroscopy. Clear guidelines for managing placental polyp are not currently available. We hope that by sharing our experience in managing such patients, especially with good outcomes, we could shed light on some of the available treatment options for these patients. 

## Conclusions

A placental polyp is a rare and enigmatic disease, which may present with various signs and symptoms, similar to other diseases. The most common and serious conditions should be explored. Tissue pathology should be obtained to ascertain a definite diagnosis. The optimal investigation and well-planned management are needed to alleviate morbidity and preserve fertility if desired. The high index of suspension should be kept as a differential as well as arteriovenous (AV) malformation. Hysteroscopy remains a good tool to reach definitive diagnosis and treatment, as in our cases. 
